# Commentary: Physical time within human time

**DOI:** 10.3389/fpsyg.2023.1101261

**Published:** 2023-06-02

**Authors:** Mark A. Elliott

**Affiliations:** School of Psychology, University of Galway, Galway, Ireland

**Keywords:** flow (dynamic), IGUS model, Aristotle, relativity, reference systems - methods, psychological time

The ideas of dimensional time, i.e., time as experienced flow, and of a zero to n-dimensional space–time are by no means new. Both ideas first appear in proto-European thought in the fragmented writings of Heraclitus in or around the fifth century BCE. The proximity of these ideas to concurrent Jewish ideas of time (related ultimately, according to some, to Mosaic teachings) suggests that the Greek and subsequently influential Latin ideas of time (particularly those expressed in Marcus Aurelias' *Meditations*) emerged from the prehistoric Egyptian mystery tradition. Ancient Egypt is known to have initiated several presocratic Greek philosophers before their return to the Hellenic world (Waterfield, 2000).

In the ancient concept of time, “experienced time” was held to exist as a past, present, and future within which changes or movements occur and time is experienced as a non-stationary present moment. This idea of time concerns the physical or, to us, the “knowable” world. In addition to the knowable, there was an infinite space–time with no beginning or end, of which only a fraction becomes experientially manifested as experienced time. Hinging upon the interpretation of Aristotle who argued in *Metaphysica* that an infinite future time could only exist in principle and, therefore, not substantially (Smith and Ross, 1908), infinite space–time was preserved in Western thought as the literal metaphysics, which was expressed in Plato's *Timaeus*. However, while expressible, we cannot, nevertheless, “know” or be able to measure this all-encompassing instance of time. Infinite time is consequently dimensionless but superordinate to time as flow, which relates more directly to the experience of the physical world and is inherently psychological. In addition, in early Jewish and the Neoplatonic and Gnostic schools of thought, although infinite time is unknowable to us, it might be “knowable” in different ways by sentient entities outside of our existential frame of reference (ultimately by God). Although not directly relevant to the topic as presented here, the importance of this idea lies in the acknowledgment that there is something other than the anthropocentric, existential “I” that has “knowledge” of time.

Our present discussion of psychological time occurs in the common scientific framework defined by physics. The entry of time into the calculus of physics could only occur post-enlightenment. Nevertheless, the idea of science is ancient and the groundwork for the entry of time into physics was laid originally by Aristotle. In *Physica*, Aristotle explicitly identified the idea of time with movement, and in turn with the flow of event structure. Important for the present discussion is the influential interpretation of this provided by Thomas Aquinas. St. Thomas clearly interpreted the Aristotelian idea of time flow as existing *only* in the experience of the soul (Hardie and Gaye, 1930; Snyder, 2000. Also note that Neoplatonist, Plotinus in Enneads stated explicitly some 1,400 years earlier that “There is for this universe no other place than the soul or mind” and “We should not accept time outside the soul or mind”, see Schopenhauer, 2000). Thus, by the late middle Ages, the eternal quality of time, while still present in cosmological theory, had become secondary to an understanding of physical time in terms of the anthropocentric and mental experience of time flow. Given his role in reconciling Christian dogma with Aristotelian logic, the influence of St. Thomas cannot be underestimated, and by virtue of his interpretation, the case is re-presented to consider time as primarily a psychological phenomenon.

For the sake of brevity, I skip past several thinkers on time from the middle Ages to the present day. In the broader context, there are thinkers on persistence through time and it is not possible to deal exhaustively with this topic in this contribution. The reader is referred to Haslanger (2003) for a review. In addition, and more specifically, there are a set of ideas considered relevant (i.e., Locke, Newton, Leibnitz, Kant, reviewed by Benjamin, 1966). These tend to concur with the idea preserved by St. Thomas that time is experienced as flow and flow concerns the mental experience of movement or change. Unfortunately, this provides us with the problem of treating time as the subject of scientific inquiry.

The problem is 2-fold: first modern theoretical physics defines experienced time as illusory because it is essentially dimensionless. This is because 4-dimensional space–time specifies that all events possess the same ontological status and are inseparable in the past, present, or future (see Poincaré, 1900; Einstein, 1905). In addition, this effective absence of dimensionality for experienced time is physical and not metaphysical. Consequently, experienced time cannot be an operational variable in the calculus of physics, and it can have no basis for consideration outside of physics either. Nevertheless, we still experience time as a non-stationary “moment now” bridging the future with the past. The problem for physics is partly retrieved by assuming that observations across a very small spatial scale will provide a measure of the experience of time flow as we know it. This is a reasonable compromise accepted by almost everyone. However, it is a compromise and the problem remains that the assumption of infinite space–time remains the province of theoretical physics, which, paradoxically, seems to prohibit an overarching and strictly scientific definition of experienced time.

Second, the problem refers to Feynman's complaint that analysis of experienced time depends on “murky notions of mentalism” (Gleick, 2011, on Feynman, 1963). Experienced time generally entails that time is experienced in the mind, and murky mentalism is another way of saying that mind–matter dualism is inadequate for scientific purposes. If we assume this to be a problem, it is (a) not resolved by empirical observation, because the observer's report of their experience is based on the mental experience and so is non-defeasible; (b) additionally, it is not resolved by correlational methods such as brain imaging, for which, brain data require a variable with *a priori* validity to correlate with; and (c) while models such as the information gathering and utilizing system (IGUS) model (Gruber et al., 2022) rely on empirical and defeasible behavioral or event data, they still rely on the reported experience of event structure to make sense in terms of experienced time flow.

However, a great deal of psychological science relies on murky mentalism, so much so that major psychological theories such as Gestalt theory premise on the validity of the phenomenal. It could be argued that the inherently non-physical defines a major remit of psychological science, which by Aristotle's definition can still be referred to as science. In the present context, approximately 2,500 years of thought on the phenomenology of time broadly concur with the idea that experienced time, including the non-stationary “now”, is valid, existent, and not illusory. This tends to suggest that the most sensible solution to the problem of the scientific definition of time is to declare the criteria set by physics to be an overreach and not appropriate for the task of explaining the experience of time.

However, this theory does not help since it does not bridge the mind–matter division. Thus, here is an alternative proposition, similar in formal structure to the IGUS model: This proposition refers to the idea that time might be experienced, and indeed the way time is processed can be measured in entities other than the existential “I”. Rather than appealing to God, I set my sights rather humbly on Elliott (2014), who showed that during the implicit coding of a repeating temporal sequence, a sequence presented so rapidly that its event structure was experienced but non-reportable, and not only was the timing of the sequence faithfully coded, but the coding mechanisms advanced in time their response to events in the sequence relative to those events. In this instance and without explicit report, or conscious experience of event structure, cognitive systems advanced their response in such a manner that event-related cognition occurred slightly ahead in time of the event to which it responded. It cannot be claimed that the observer has conscious access, that they can report anything as experienced by the “I”, or that their first-person experience of derivative events occurs in future time. However, this evidence nevertheless shows that experience *in the receiver* can operate in future time, and to make this claim, one must adopt the position that in order to do so, it is the system as an “entity” that experiences events in future time, and consequently, event structure is separated into past, present, and future [For a related discussion based on the role of neural oscillation in perception, the reader referred to communication through coherence (CTC) theory by Fries, 2015].

## Conclusion

In conclusion, science need not throw out the baby with the proverbial bathwater. Instead, the variables used to define temporal experience need to be examined carefully and broadened appropriately and not put into a conceptual frame of reference to which they do not fit. Of course, this is a problem for the strictest definition of science, but not necessarily for psychology. Psychological science might accept that it occupies a position that is a challenge to this strictly reductionist scientific agenda, and it might be content to define its own validity regardless. In this enterprise, there has been consistent support on what defines the experienced time for a very long time.

## Author contributions

The author confirms being the sole contributor of this work and has approved it for publication.
